# Introgression of wild barley alleles improves seedlings salinity tolerance in the nested association mapping HEB‐400 population

**DOI:** 10.1002/tpg2.70217

**Published:** 2026-03-15

**Authors:** Matías Schierenbeck, Radwa Y. Helmi, Andreas Maurer, Rasha A. Tarawneh, Doaa H. Ali, Hannah M. Schneider, Andreas Börner, Klaus Pillen, Helmy M. Youssef

**Affiliations:** ^1^ Leibniz Institute of Plant Genetics and Crop Plant Research (IPK) Seeland Germany; ^2^ Genetics and Cytology Department Biotechnology Research Institute National Research Centre Giza Egypt; ^3^ Institute of Agricultural and Nutritional Sciences Martin‐Luther‐University Halle‐Wittenberg Halle Germany; ^4^ Faculty of Agricultural Technology Al‐Ahliyya Amman University Amman Jordan; ^5^ Plant Biotechnology Department Biotechnology Research Institute National Research Centre Giza Egypt; ^6^ Faculty of Agriculture Cairo University Giza Egypt; ^7^ Research, Development and Innovation Centre for Converging Science and Emerging Technology (RDI CoSET) Benha National University El‐Obour City Egypt

## Abstract

Climate change is intensifying the frequency and severity of abiotic stresses that threaten global food security by reducing crop productivity. Among these, saline stress poses a serious threat to barley (*Hordeum vulgare* L.) production. These conditions are increasingly prevalent in arid and semiarid regions, as well as in regions with limited access to freshwater resources, making the identification of salt tolerance genes essential for breeding resilient varieties. In this study, we evaluated 400 genotypes from the barley nested association mapping population HEB‐25 under control conditions and 40% seawater irrigation to simulate moderate‐to‐high salinity stress. A genome‐wide association study (GWAS) was conducted to identify alleles from wild barley [*H. vulgare* L. subsp. *spontaneum* (C. Koch) Thell.] associated with enhanced salt tolerance. Phenotypic evaluation included germination percentage (Ger%), shoot length (SL), root length (RL), root–shoot length ratio, seedling fresh weight, seedling dry weight, and salt tolerance index of the different traits. The HEB‐25 families exhibited significant variation in seedling responses to seawater‐induced salinity, with contrasting effects on SL, RL, and dry weight. Compared to the elite parental Barke, several genotypes demonstrated high tolerance under seawater stress, maintaining stable Ger% and exhibiting the highest tolerance indices. Moreover, GWAS results identified 60 highly significant single nucleotide polymorphisms associated with seedling growth parameters under both conditions. These findings underscore the value of the HEB‐400 panel as a genetic resource for dissecting salinity tolerance mechanisms, identifying stress‐adaptive alleles lost during domestication and a source of pre‐breeding material for developing genotypes with enhanced salinity tolerance.

## Core Ideas

- Some wild barley alleles improved seedling growth tolerance traits under saline stress.
- Several HEB lines outperformed the elite cultivar Barke under saline conditions.
- Genome‐wide association study (GWAS) in HEB‐400 panel identified 60 quantitative trait loci (QTLs) for seedling traits under moderate‐to‐high salinity stress.
- Candidate genes associated with ion transport, signaling pathways, and stress adaptation were reported.
- The HEB‐400 panel provides valuable pre‐breeding germplasm with improved salinity tolerance.

## Plain Language Summary

Climate change is increasing soil salinity in farmlands worldwide, threatening barley production. While modern barley varieties have lost some natural resilience during domestication, their wild relatives retain valuable stress‐tolerant genes. This study explored how introducing genes from wild barley (*Hordeum vulgare* ssp. *spontaneum*) into elite barley varieties to enhance their ability to thrive in saline soils.

**Abbreviations**
CL: coleoptile length
DW: dry weight
DW/FW‐R: DW/FW ratio
FW: fresh weight
Ger%: germination percent
GWAS: genome‐wide association study
QTL: quantitative trait loci
RL: root length
RSR: root–shoot length ratio
SL: shoot length
SNP: single nucleotide polymorphism
TI: tolerance index
WCP: water content percentage

## 1 INTRODUCTION

Climate change is a major driver of global abiotic stress, significantly affecting agricultural land and hindering plant growth and development (Chowdhury et al., 2025; Porter et al., 2019). Abiotic factors such as salinity and drought have been widely studied for their impact on plant productivity through the suppression of key metabolic processes (Almaghrabi, 2012; Raza et al., 2022; Villalobos‐López et al., 2022; P. Yuan, Usman, et al., 2024; X. Yuan, Li, et al., 2024). Globally, approximately 20% of cultivated lands and 33% of irrigated lands are affected by salt stress (Hanafy et al., 2022). The rate of soil salinization is estimated to be 1–2 million ha per year, which has a significant impact on crop production and renders a substantial area of land unsuitable for cultivation. Salinity has been shown to reduce crop yield in two main ways: first, it inhibits plant access to soil water by increasing its osmotic potential, thereby limiting the availability of water and nutrients (osmotic or water‐deficit effect). Second, it causes ionic imbalance and toxicity in plants (salt‐specific or ion‐excess effect) (Zhang et al., 2023). The effect of salinity on crop growth is stage‐dependent, with germination and early seedling stages showing greater sensitivity than later growth stages (Akinnagbe & Irohibe, 2015; Schierenbeck et al., 2023). This differential sensitivity has implications for plant development and yield productivity (Rajesh et al., 2024). Strategies to mitigate the impact of salinity on soil and crop production include the use of desalinated irrigation water, soil leaching, improved irrigation techniques, enhanced field drainage, and the cultivation of salt‐tolerant or newly developed crop varieties (Singh et al., 2024). Currently, growing adapted and newly developed cultivars is the most economical and efficient way to minimize grain yield reductions under saline stress conditions (Karunarathne et al., 2023). Developing adapted cultivars of key crops requires an understanding of the physiological mechanisms and the genetic basis underlying different stages of plant development (Akula & Ravishankar, 2011).

Barley (*Hordeum vulgare* L.) is one of the most important cereal crops, along with wheat (*Triticum aestivum* L.), maize (*Zea mays* L.), and rice (*Oryza sativa* L.) (Youssef et al., 2020). In addition to its capacity to thrive in diverse environmental conditions, it is recognized as one of the most resilient cereal crops, enabling its growth at extreme latitudes and altitudes where other crops are unable to adapt (Moshelion & Altman, 2015). Barley's ability to thrive in saline environments is well documented (Dias, 2015), and its adaptation capabilities have led to its use as a standard model plant in breeding programs aimed at improving other cereals (Munns & Tester, 2008). Over the last century, selective breeding processes have resulted in significant erosion of barley genetic resources, thus reducing the genetic diversity among its cultivars compared to ancestral [*H. vulgare L*. subsp. *spontaneum* (C. Koch) Thell.]. Reduced biodiversity results in poor fitness and yield instability under adverse conditions as reported in different crops (Bruce et al., 2002; El‐Mahrouk et al., 2025; Russell et al., 2016).

The molecular mechanisms underlying salinity tolerance in barley involve complex traits regulated by multiple quantitative trait loci (QTLs) and genes associated with ion homeostasis, osmotic adjustment, and stress signaling (Munns & Tester, 2008; Nevo & Chen, 2010). Several QTLs for salinity tolerance have been mapped in barley, particularly on chromosomes 2H, 4H, and 7H. A major QTL on chromosome 7H has been consistently associated with shoot Na^+^ exclusion and improved growth under saline conditions (Fan et al., 2016; Xue et al., 2017). Genes such as *HvHKT1;5*, a member of the high‐affinity potassium transporter family located on chromosome 4H, have been functionally characterized and shown to mediate Na^+^ retrieval from the xylem, reducing toxic accumulation in the shoot (Huang et al., 2020). Additionally, *HvNHX1*, a vacuolar Na^+^/H^+^ antiporter, contributes to intracellular ion compartmentalization and osmotic balance under salt stress (Fukuda et al., 2004).

Other candidate genes implicated in salinity tolerance include members of the SOS (salt overly sensitive) pathway, such as *HvSOS1*, which facilitates Na^+^ efflux at the plasma membrane, and transcription factors like *HvDREB1* and *HvWRKY*, which modulate the expression of downstream stress‐responsive genes (Shavrukov, 2013). Integrative approaches combining biparental QTL mapping, genome‐wide association studies (GWASs), and transcriptomic profiling significantly advanced our understanding of the complex genetic architecture underlying salinity tolerance in this crop. These strategies have enabled the identification of both major and minor loci, facilitated the discovery of candidate genes with regulatory and transporter functions, and provided insight into genotype‐specific expression patterns under salt stress (Saade et al., 2016). Such multilayered analyses are instrumental in linking phenotypic variation to specific genetic mechanisms, thereby informing marker‐assisted selection and the development of salt‐tolerant cultivars.

Therefore, revisiting the potential of the cultivated gene pool and incorporating untapped barley genetic diversity is a major challenge for barley sustainability (Bapela et al., 2022; Capasso et al., 2021). For this reason, a nested association mapping (NAM) barley population (HEB‐25) was developed by crossing and backcrossing a diverse set of 25 wild progenitors with an elite barley cultivar to investigate exotic allele introgression effects in an elite background (Maurer et al., 2015).

The early stages of the barley life cycle—germination and seedling development—are especially vulnerable to seawater irrigation conditions and are considered critical for determining grain yield (Tarawneh et al., 2020). Accordingly, the development of new cultivars that tolerate salinity at these early stages is essential in regions that are affected by dry summers, which increases salinity levels in the topsoil (Jalil & Ansari, 2020). There is a lack of knowledge about the genetic basis of early seedling salinity tolerance under seawater treatment conditions, as little research has been conducted to characterize the responsible genes and identify marker–trait associations (MTAs) regulating their performance under saline stress. Unlike previous studies that simulated salinity stress using sodium chloride (NaCl) solutions alone (Munns & Tester, 2008; Roy et al., 2014), our use of seawater provides a more ecologically relevant and ionically complex representation of saline conditions and better reflecting the challenges faced in natural saline soils and coastal agricultural environments.

Recently, using the high‐throughput genotyping based on single nucleotide polymorphism (SNP) arrays or progress of DNA sequencing technology and next‐generation sequencing, GWASs of complex quantitative traits have become an advanced tool of genetic studies (Mondal et al., 2021). GWAS analysis helped unravel the genetic basis of genes associated with salinity‐related traits in crops such as wheat (Hasanuzzaman et al., 2022; Ncama et al., 2022; Sayed et al., 2021). Linkage mapping studies have further validated several genomic regions/MTAs detected by GWAS for different traits (Sayed et al., 2022; Takeda & Matsuoka, 2008). Thus, the overall objective of this study was to use GWAS to identify salinity‐related QTLs and potential candidate genes playing a role in the regulation of germination and seedling traits in a panel of 400 genotypes selected from the barley NAM population HEB‐25. Due to the inclusion of exotic alleles, this population has been previously utilized in diverse studies including flowering time, grain yield, drought tolerance, grain quality traits, and fungal disease resistance under diverse environments (Büttner et al., 2020; Herzig et al., 2018; Maurer et al., 2015, 2016; Pham et al., 2019, 2024; Saade et al., 2016; Sharma et al., 2018; Vatter et al., 2017). The use of this population offers a unique advantage for dissecting salinity tolerance at the seedling stage, as it enables the evaluation of wild allele introgressions in a controlled elite background, facilitating the identification of genomic regions associated with early‐stage salt stress adaptation.

**Core Ideas**

- Some wild barley alleles improved seedling growth tolerance traits under saline stress.
- Several HEB lines outperformed the elite cultivar Barke under saline conditions.
- Genome‐wide association study (GWAS) in HEB‐400 panel identified 60 quantitative trait loci (QTLs) for seedling traits under moderate‐to‐high salinity stress.
- Candidate genes associated with ion transport, signaling pathways, and stress adaptation were reported.
- The HEB‐400 panel provides valuable pre‐breeding germplasm with improved salinity tolerance.

## 2 MATERIALS AND METHODS

### 2.1 Plant materials

The germplasm used in this study consisted of 400 BC1S3 lines (derived from one backcross followed by three generations of selfing) that were selected from the multi‐parental barley NAM population Halle Exotic Barley (HEB‐25) and named as HEB‐400. The HEB‐400 subset was selected based on three main criteria. The lines should have no brittle rachis and be easily threshed, while also representing the genetic diversity of the entire population. More details of this subset can be found in Maurer et al. (2015).

For clarity, the panel of 400 genotypes used in the present study will hereafter be referred to as HEB‐400. The designation HEB‐25 is reserved for the original HEB‐25 population.

### 2.2 Genotyping of HEB‐400 lines

DNA extraction and HEB‐400 genotyping are described in Gemmer et al. (2020). DNA was extracted from eight plants of each line pooled together from BC1S3 using the BioSprint 96 DNA Plant Kit and a BioSprint workstation (Qiagen), and then dissolved in distilled water at approximately 50 ng/µL. The genotyping of the population was carried out using the Infinium iSelect 50K SNP chip (Bayer et al., 2017) at Trait Genetics. SNP markers that did not meet the quality criteria (polymorphic in at least one HEB family, <10% failure rate, <12.5% heterozygous calls) were removed from the dataset. In total, 32,995 SNPs were used in this study. The genotype matrix was translated into a quantitative 0‐1‐2 matrix (0 = Barke allele, 1 = heterozygous, 2 = wild barley allele) and is available at e!DAL (Maurer & Pillen, 2021). Population structure was estimated through principal coordinate analysis (SAS PROC PRINCOMP) based on a genetic similarity matrix (simple matching) applied on the whole SNP dataset (SAS PROC DISTANCE) (Figure S1). Furthermore, to estimate linkage disequilibrium (LD) the R package “sommer” was utilized to calculate intrachromosomal *r* ^2^ of marker pairs with the function “LD.decay.” LD decay was defined by fitting a LOESS (locally estimated scatterplot smoothing) curve based on the pairwise physical distance obtained from Morex RefSeq2 (Monat et al., 2019) and determining the distance when the LOESS fit crossed the *r* ^2^ threshold of 0.2 (Figure S2).

### 2.3 Growing conditions and germination and seedling traits

This experiment was conducted in a complete randomized design at the Leibniz Institute of Plant Genetics and Crop Plant Research (IPK), Gatersleben, Germany. A set of seeds of each line was sterilized using 70% ethanol solution for 1 min, rinsed with sterile distilled water three times, then briefly blotted. For each repetition, 16 seeds per line were germinated according to the International Seed Testing Association protocol (ISTA, 2013). Blotting paper sheets of 25 cm × 60 cm (Ahlstrom Munnksjö GmbH) were used in this experiment. Seeds were positioned approximately 5 cm from the top of the wet sheet, using tap water for the control and 40% diluted Mediterranean microfiltered saline water (23.2 g/L [Cl], 9.8 g/L [Na], 0.359 g/L [Mg], 3.4 g/L [SO_4_], 0.409 g/L [Ca], 0.465 g/L [K], 0.144 g/L [Bicarbonates], <2.4 mg/L [Boron], salt content 36.0 g/L, pH 7.8) in tap water for the salinity treatment. The 40% seawater concentration was selected to simulate realistic conditions where saline or partially saline water is used for irrigation (e.g., in newly reclaimed areas in Egypt and Jordan where freshwater availability is limited). Then, the seeds were covered with another wet sheet and rolled to separate the seeds from each other. The control (C) and salinity treatment (S) sheets were placed in plastic bags and watered with tap water or diluted seawater for the respective treatments. Both control and salinity treatment bags were placed in a versatile test chamber (Model No. MLR‐352‐PE, Panasonic) for three days at 20 ± 2°C in dark conditions and then moved to the same type of test chambers for 7 days at 20 ± 2°C and 16 ± 2°C with 16 h day/8 h night photoperiod. A total of four independent biological replicates were conducted for each line under each treatment condition.

After 10 days of incubation, the number of germinated seeds (>3 mm radicle) was counted. The germination percentage (Ger%) was calculated for each accession: Ger % = seeds germinated/total seeds × 100. Germination tolerance index (Ger_TI) was calculated as: Ger_TI = Ger % under salt treatment/Ger % under control. The seedling growth parameters were measured as follows: coleoptile length (CL), shoot length (SL), and longest root length (RL) were manually measured at the 10th day of germination using a scaled ruler for eight seedlings per repetition within each genotype and treatment. Root–shoot length ratio (RSR) was calculated as the ratio of the RL to the SL. The seedling's fresh weight (SFW) was recorded (g) using an ultra‐micro lab balance (Sartorius AC 1215), then seedlings were dried at 60°C for 72 h to obtain the seedling dry weight (SDW). Water content percentage (WCP) was calculated based on the following formula: WCP (%) = [(SFW − SDW)/SFW] × 100. Trait measurements under control (C) and salt stress (S) conditions were denoted using suffixes (e.g., _C, _S). For instance, CL_C and CL_S refer to coleoptile length under control and salt stress, respectively. A tolerance index (TI) was defined for all the traits as the ratio of the mean value under stress to that under control conditions (e.g., TI_SL = SL_S/SL_C).

### 2.4 ANOVA analysis of phenotypic data

An analysis of variance (ANOVA) was conducted to compare genotypes and traits using GENSTAT for Windows Ver. 19 (VSN International) for the seedling growth traits. The probability of significance in ANOVA (*p* ≤ 0.05) was used to indicate significant differences among genotypes (G), treatments (T), and interaction effects (G × T). Means were separated according to Fisher's least significant difference *p* ≤ 0.05 levels of probability. Broad‐sense heritability (*H* ^2^) was estimated using variance components derived from GenStat 19 software, based on the following model:

$$Y_{\left(ijk\right)}=\mu +G_{i}+T_{j}+{\left(GT\right)}_{ij}+R_{k}\left(T_{j}\right)+\varepsilon_{\left(ijk\right)}$$

where *Y_ijk_* ​ is the observed value of the *i*th genotype in the *j*th treatment and *k*th replicate, *μ* is the overall mean, *G_i_​* is the random effect of the *i*th genotype, *T_j_​* is the fixed effect of the *j*th treatment, (*GT*)*_ij_* is the genotype × treatment interaction, *R_k_* (*T_j_*) is the random effect of the *k*th replicate nested within each treatment, and *ε_ijk_* ​ is the residual error.

Broad‐sense heritability was then calculated as:

$$H^{2}=\sigma g^{2}/\left[\sigma g^{2}+\left(\sigma gy^{2}/y\right)+\left(\sigma e^{2}/\left(y\times r\right)\right)\right]$$

where (*σg* ^2^) are the estimated variance components, genotype × treatment interaction (*σgy^2^*), residual error (*σe* ^2^), and *y* represents treatment and *r* (replicates). MVApp v2.0 (Julkowska et al., 2019) was used for correlation and SRplot boxplot calculations (Tang et al., 2023). The restricted maximum likelihood algorithm was applied and the best linear unbiased estimators (BLUEs) at the genotype level within each treatment were calculated using the nlme package in R (Pinheiro et al., 2025). BLUEs were obtained from the mixed‐model **β^​ = (*X*′*V* ^−1^ *X*)^−1^ *X*′*V* ^−1^ *y***, where ***y*** is the phenotypic vector, ***X*** is the design matrix for fixed effects, ***V*** is the variance–covariance matrix of the model, and ***β^*** ​ is the resulting vector of BLUEs.

### 2.5 GWAS and candidate gene detection

A GWAS was performed with a total number of 32,995 polymorphic SNPs in an identity‐by‐state (IBS) matrix (matrix E) from Maurer and Pillen (2021). The analysis followed a robust three‐step procedure that was implemented in SAS 9.4 (SAS Institute Inc.), as described in Dreissig et al. (2020). (I) The selection of associated SNPs was conducted using a multiple linear regression model encompassing all SNPs, implemented via SAS PROC GLMSELECT. The process involved the creation of 100 repeated subsamples, each comprising 80% of the dataset. Only those SNP markers that contributed to the prediction of the remaining 20% were selected, with the selection criterion being the minimization of the average squared error, computed as the sum of squared errors divided by the number of observations (*N*). SNPs that were selected more than once in this initial step were identified as potential cofactors. (II) These potential cofactors were subsequently used as input for a more refined cofactor selection using SAS PROC GLMSELECT, with the Schwarz Bayesian criterion (Schwarz, 1978) set to a minimum, applied to the entire dataset. (III) Following this, each individual SNP was screened for significance through multiple linear regression, with the previously identified cofactors included in the model background (in the order of their model inclusion from step II). This step was performed using SAS PROC REG, with squared semi‐partial correlation coefficients (*R* ^2^) generated using the SEQTESTS model option. This method is recognized as highly robust for defining significant SNPs, as it substantially reduces the risk of model overfitting. Furthermore, the *R* ^2^ values, *p* values, and allele effects were estimated in the presence of the selected cofactors. Candidate genes were identified based on the physical position of significant SNPs, anchored to the Morex v2 reference genome, using the BARLEX database (https://apex.ipk‐gatersleben.de/apex/f?p=284:10). To account for the genetic resolution of the HEB‐400 population and the marker density of the 50K SNP chip, we defined candidate regions as 1.7 Mbp windows surrounding each significant SNP (Figure S2). This threshold is consistent with previous GWAS studies in barley employing similar populations and genotyping platforms, which report LD decay ranging from approximately 200 kb up to 1 Mb, depending on the genomic region and recombination landscape (Maurer et al., 2015; Wiegmann et al., 2019). Genes within these windows were extracted using BARLEX and further characterized based on their gene annotation, molecular function, and biological processes.

## 3 RESULTS

### 3.1 Seawater stress effects and tolerance variation among HEB‐25 families at the seedling stage

**FIGURE 1:**
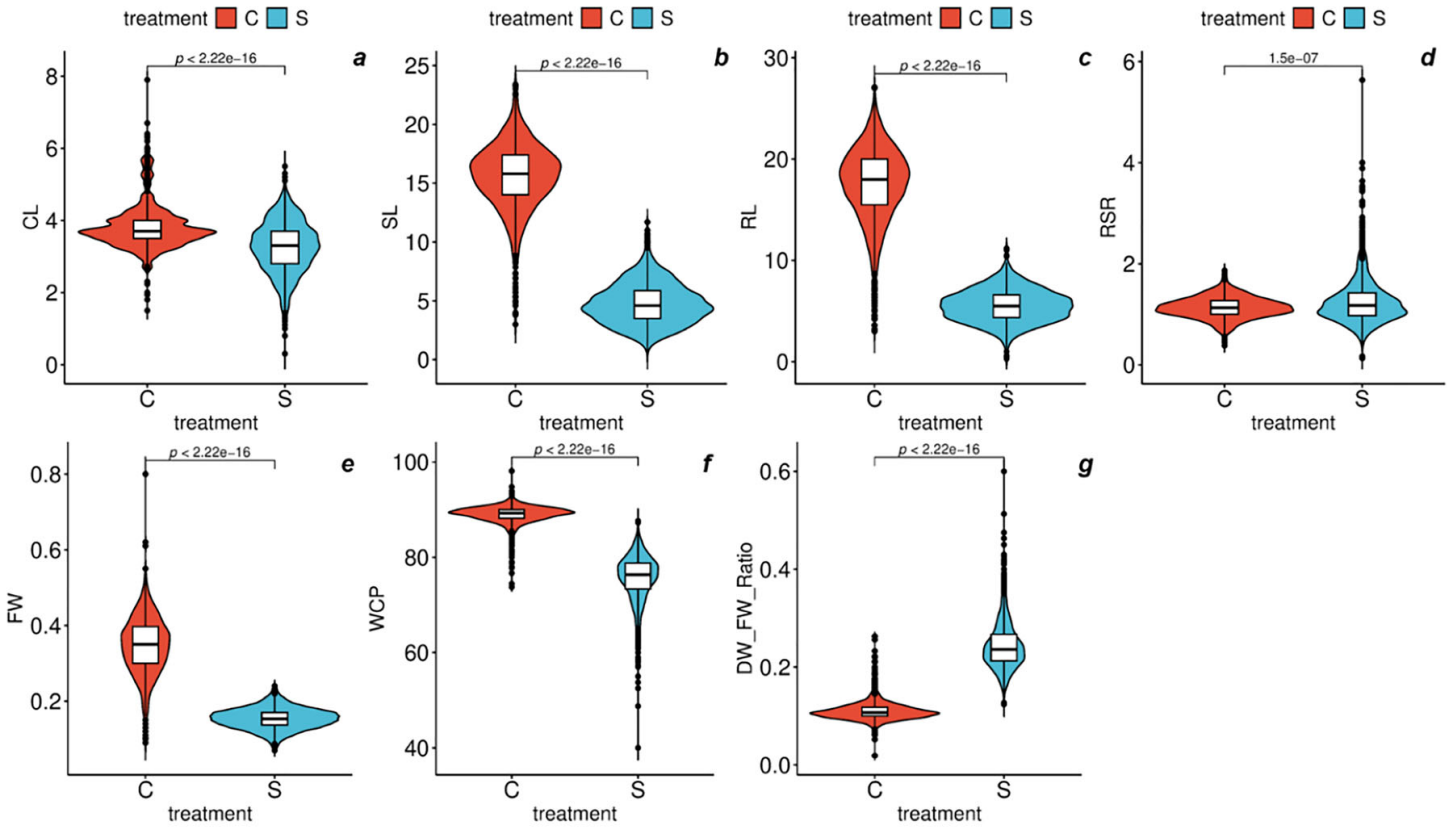
Violin and Boxplots for seedling growth traits of the barley HEB‐400 population under control (C) and seawater (40%) (S) treatments. (a) Coleoptile length (CL in cm), (b) shoot length (SL in cm), (c) root length (RL in cm), (d) root–shoot length ratio (RSR), (e) fresh weight (FW in g), (f) water content percentage (WCP), and (g) dry weight to fresh weight ratio (DW/FW‐R). Violin plots represent data distributions based on 3200 observations (1600 per treatment) across 400 genotypes.

Seawater treatment negatively affects overall seedling growth by reducing SL and RL, and SFW (Figure 1). However, plants show adaptive responses like increased RSR and tissue water content under stress. Compared to the control, seawater treatment led to reductions (averaged across genotypes) of 15.1% in CL, 69.5% in SL, 68.4% in RL, 57.1% in fresh weight (FW), and 15% for WCP. In contrast, increases were observed in the RSR (11.5%) and in the dry weight (DW)/FW ratio (118.2%). Nonsignificant reductions were reported for Ger % and DW (Table S1).

**FIGURE 2:**
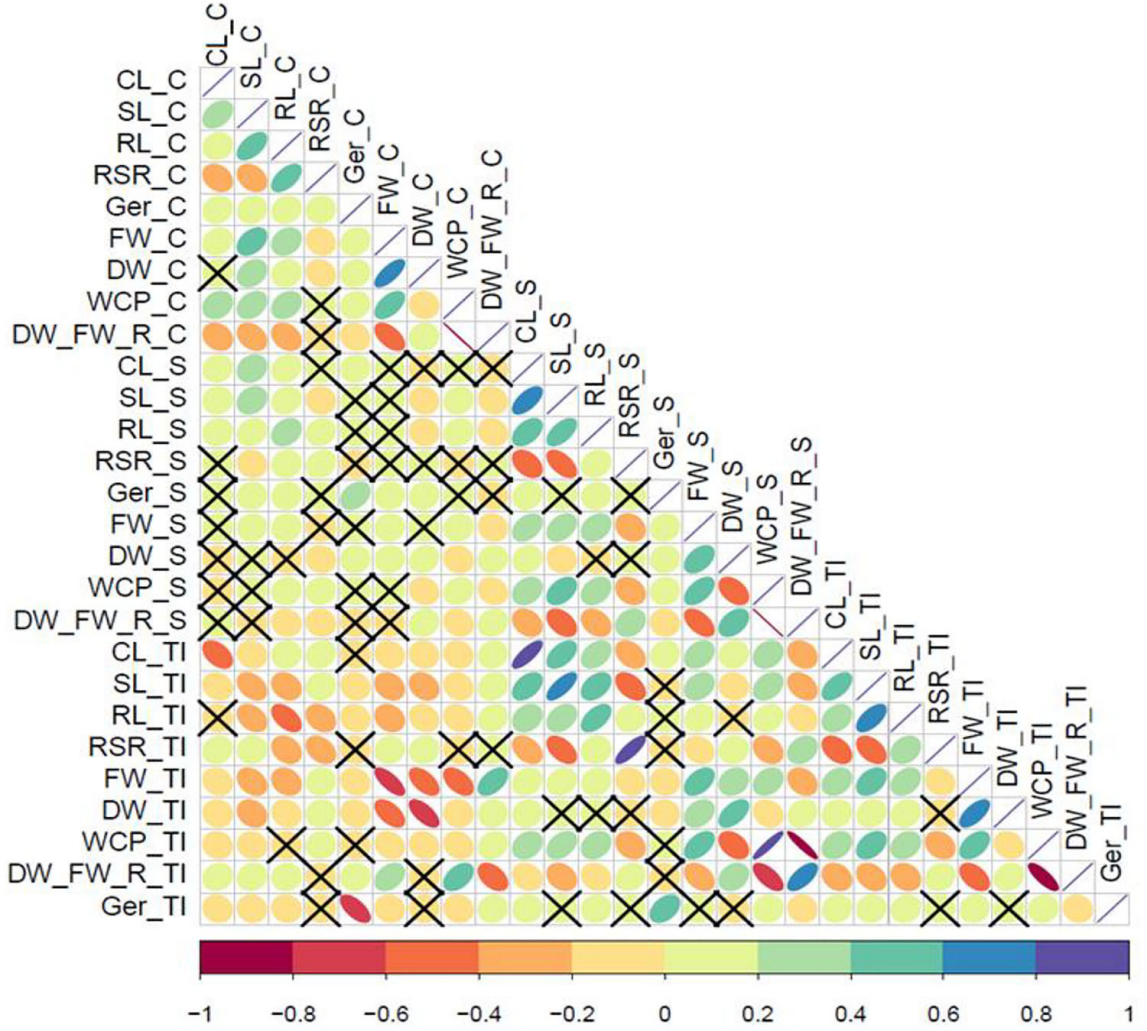
Pearson correlation coefficients among seedling growth parameters under control and seawater stress conditions for the HEB‐400 panel. Treatments: control (C), seawater 40% (S), and tolerance index (TI). Seedling traits scored: coleoptile length (CL), shoot length (SL), root length (RL), root–shoot length ratio (RSR), germination percentage (Ger), fresh weight (FW), dry weight (DW), water content percentage (WCP), and dry to fresh weight ratio (DW/FW‐R). The degree of significance for all correlations was *p* ≤ 0.001. The color reflects the strength of the correlation. Black crosses indicate nonsignificant correlations.

A highly positive correlation was reported between the RSR_TI and RSR_S (*r* = 0.82), CL_TI and CL_S (*r* = 0.80), and WCP_TI with WCP_S (*r* = 0.94). Furthermore, a positive correlation was observed between FW_C and DW_C (*r* = 0.75), the DW/FW‐R_TI and DW/FW‐R_S (*r* = 0.66) (where DW/FW is the dry weight to fresh weight ratio), the DW_TI and FW_TI (*r* = 0.67), the SL_TI and SL_S (*r* = 0.75), and the SL_TI with RL_TI (*r* = 0.64). High negative correlations (*r* = −0.81 to −0.99) were detected between DW/FW‐R and WCP under control, seawater stress, and TI. Negative correlations were also found between DW_C and DW_TI (*r* = −0.67), the FW_C and FW_TI (*r* = −0.74), and the DW/FW‐R_TI and WCP_S (*r* = −0.66) (Figure 2; Figure S3).

With exception of %Ger and DW, all studied traits were significantly influenced by genotypes, treatments, families, and their interactions (Table S1). Data analysis revealed extensive phenotypic variation in all the studied traits. Broad‐sense heritability was high, ranging from 0.791 to 0.917 for the different seedling growth parameters under control conditions, from 0.760 to 0.866 under seawater stress, and from 0.690 to 0.826 for TI traits (Table S1). The highest heritable trait was CL under the control condition (0.917), while DW TI showed the lower heritability (0.690).

**FIGURE 3:**
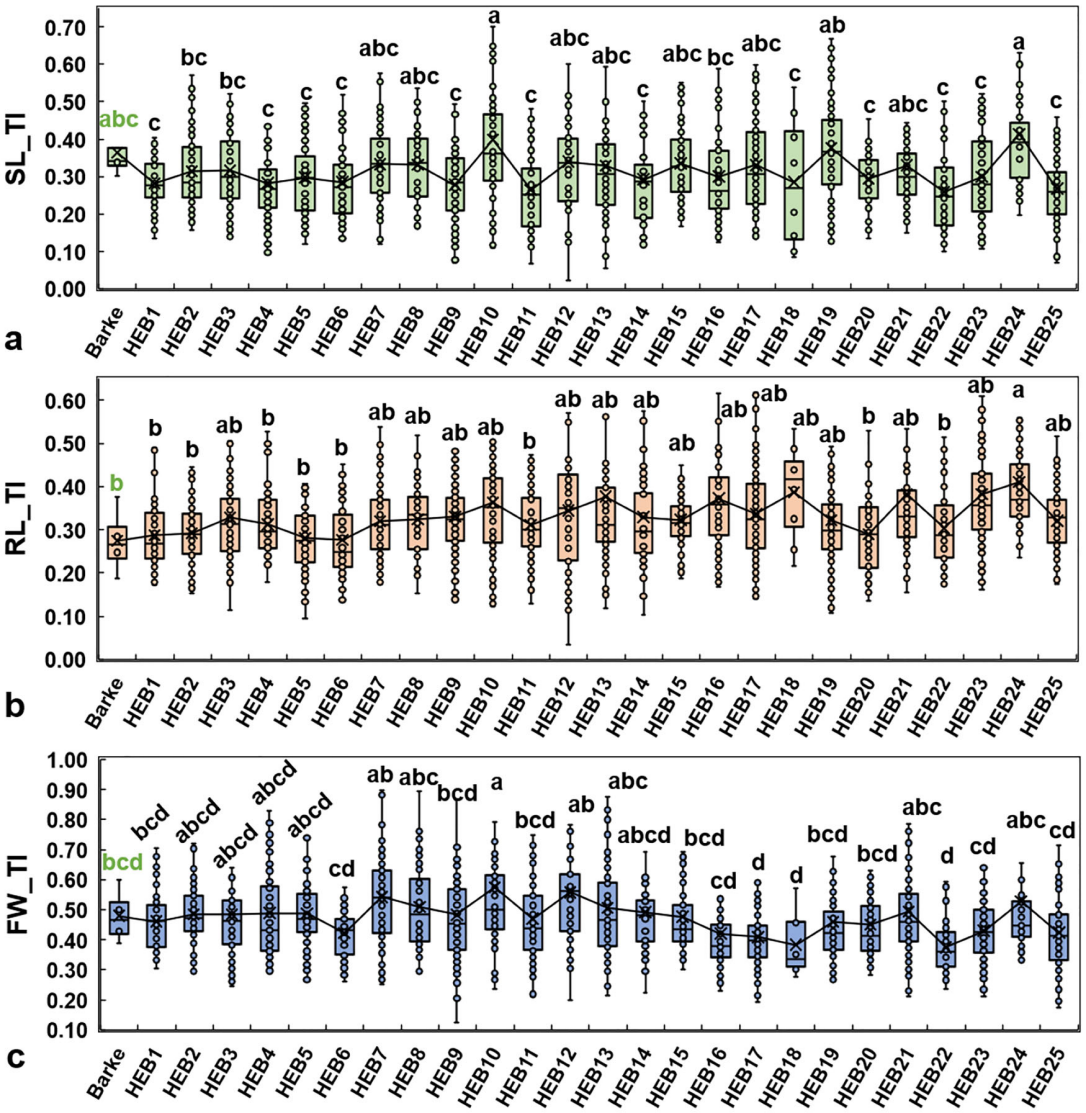
Boxplot showing tolerance index means for (a) shoot length (SL), (b) root length (RL), and (c) seedling fresh weight (FW) among the HEB‐25 families and the elite cultivar Barke (in green). Data points of each boxplot represent the genotype means of a given HEB family. Error bars, outliers, median, and mean values (crosses) are included. Means followed by the same letter are not statistically different (least significant difference (LSD) *p* ≤ 0.05).

Significant variations were observed among HEB‐25 families’ seedling growth TI compared to the elite cultivar Barke. Differences were detected across several traits, including SL and RL, and FW and CL (Figure 3; Tables S1–S4).

Analyses of the HEB‐25 population revealed considerable variation in TI means across families for SL (SL_TI), RL (RL_TI), FW (FW_TI), and CL (CL_TI) (data not shown). Among these families, HEB10 (FW_TI, CL_TI, SL_TI, and RL_TI) and HEB24 (FW_TI, RL_TI, and SL_TI) consistently displayed the highest mean values, suggesting a higher saline stress tolerance (Figure 3). In contrast, families such as HEB25, HEB22, and HEB20 ranked among the lowest across most traits, indicating reduced tolerance. Interestingly, the elite parent Barke showed good performance, especially for SL_TI and CL_TI, but was outperformed by several HEB lines in RL_TI (0.273) (Table S3).

### 3.2 GWAS identified multi‐trait QTLs and genomic hotspots for salinity tolerance in selected genotypes from the HEB‐400 barley panel

**FIGURE 4:**
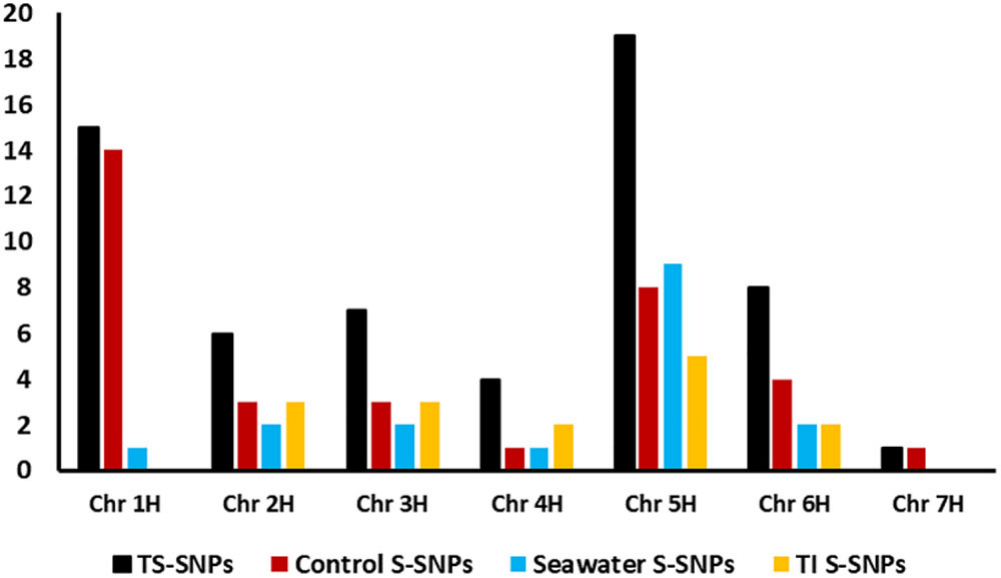
Total number of significant single nucleotide polymorphisms (SNPs) (TS‐SNPs), significant SNPs under control and seawater conditions, and tolerance index (TI) significant SNPs (TI S‐SNPs) distributed on the seven barley chromosomes.

**FIGURE 5:**
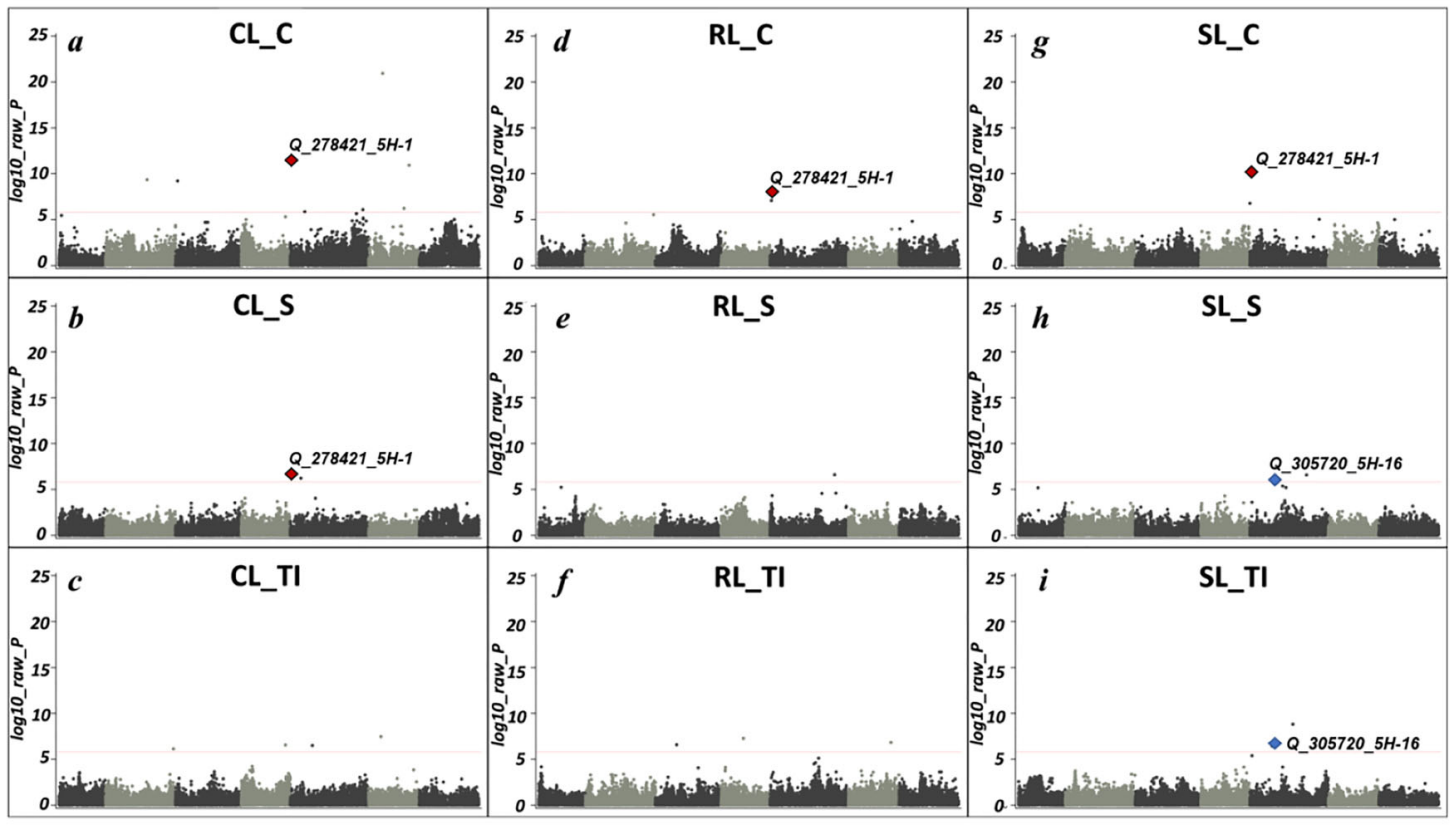
Manhattan plots (a–i) showing the distribution of quantitative trait loci (QTLs) associated with coleoptile length (a–c), root length (d–f), shoot length (g–i) under control conditions, seawater conditions, and tolerance index for the 400 barley HEB lines. QTLs are ordered based on Morex RefSeq2 (Monat et al., 2019). Multi‐traits QTLs *Q_278421_5H‐1* (red) and *Q_305720_5H‐16* (blue) are marked with colored dots. The red dashed line indicates the Bonferroni threshold of significance at *p* ≤ 0.05 based on 32,995 single nucleotide polymorphisms (SNPs) (logarithm of odds ≥  5.82).

The GWAS analysis identified a total of 60 QTLs (logarithm of the odds  ≥ 5.82) related to germination and diverse seedling growth parameters under control, seawater treatment, and their TIs. Interestingly, chromosome 5H showed the largest proportion of QTLs (19 QTLs), while 1H (15), 6H (8), 3H (7), 2H (6), 4H (4), and 7H (1) exhibited a lower number of associations (Figures 4 and 5; Figure S4; Table S5). A total of 19, 26, and 10 QTLs were associated with traits under control treatment, under seawater treatment, and TIs, respectively. In total, five QTLs were co‐localized between the control and salinity treatments and their TIs (Table S5).

Our analysis revealed that the number of QTLs was generally higher under control treatment than under seawater treatment for most of the traits. However, WCP and DW/FW‐R showed more QTLs under seawater treatment (sixteen and three, respectively) than under control (two and two). For SL, RSR, and DW, the number of QTLs was the same in both conditions. In contrast, very few QTLs were detected for the salt TIs: one QTL for DW/FW‐RTI and four for CL_TI (Figures 4 and 5; Table S5; Table 1). A complete list of all QTLs, including marker information, chromosomal positions, associated traits, and allelic effects is provided in Table S5.

**TABLE 1:** Total number of quantitative trait loci (QTLs) (TS‐QTLs) detected in the study under control and seawater stress treatments.

Traits	No. TS‐QTLs	QTLs under control	QTLs under seawater	QTLs under control and seawater	Positive Hsp effects	Negative Hsp effects	QTLs on chr. 1H	QTLs on chr. 2H	QTLs on chr. 3H	QTLs on chr. 4H	QTLs on chr. 5H	QTLs on chr. 6H	QTLs on chr. 7H
CL	9	7	1	1	7	2	0	1	1	0	4	3	0
SL	4	2	2	0	1	3	0	0	0	0	4	0	0
RL	3	2	1	0	1	2	0	0	0	0	3	0	0
RSR	10	5	5	0	9	1	0	3	1	0	5	1	0
Ger%	5	3	2	0	0	5	0	0	0	0	3	1	1
FW	2	2	0	0	0	2	0	0	0	1	1	0	0
DW	4	2	2	0	2	2	0	0	1	2	1	0	0
WCP	19	2	16	1	0	19	14	1	2	0	1	1	0
DW/FW‐R	6	2	3	1	6	0	2	1	2	0	1	0	0
CL_TI	4	0	0	0	0	4	0	1	0	1	1	1	0
SL_TI	2	0	0	0	1	1	0	0	0	0	2	0	0
RL_TI	3	0	0	0	3	0	0	0	1	1	0	1	0
R/S‐R_TI	3	0	0	0	3	0	0	1	1	0	1	0	0
FW_TI	2	0	0	0	2	0	0	0	1	0	1	0	0
DW_TI	2	0	0	0	2	0	0	0	1	0	1	0	0
WCP_TI	3	0	0	0	1	2	0	2	0	0	1	0	0
DW/FW‐R_TI	1	0	0	0	1	0	0	0	1	0	0	0	0

*Note*: Positive and negative Hsp effects indicate the genotypes average difference of phenotypes between wild barley and elite barley alleles at a QTL.

Abbreviations: CL, coleoptile; DW, dry weight; DW/FW‐R, DW/FW ratio; FW, fresh weight; Ger%, germination percent; Hsp, *Hordeum vulgare* ssp. *spontaneum* (wild barley); RL, root length; R/S‐R, root shoot ratio; SL: shoot length; TI: tolerance index; WCP, water content percent.

Following the identification of these key QTLs, further analysis revealed 60 high‐confidence candidate genes (Table 2; Table S5). Of these, 41 genes were detected under control conditions and 34 genes were identified under seawater treatment, with the latter being associated with various seedling growth traits.

**TABLE 2:** List of single nucleotide polymorphism (SNP) markers associated with multiple traits simultaneously and their closest high confidence genes.

No.	Trait	SNP marker	Chr.	Refseq2.0 gene	QTL‐ID	Annotation inRefseq2.0	Bon_p
1	DW_FW_R_C; WCP_C; FWTI; CL_C; SL_C; RSR_C; DW_FW_R_S; RL_C; WCP_TI; WCP_S; CL_S; FW_C	JHI_Hv50k_2016_278421	5H	HORVU.MOREX.r2.5HG0350240	Q_278421_5H‐1	Peroxisome biogenesis protein 3‐2	1.3917E‐^80^
2	WCPTI; CL_TI	JHI_Hv50k_2016_143769	2H	HORVU.MOREX.r2.2HG0178720	Q_143769_2H‐3	Cysteine proteinase inhibitor	0.000151
3	RSR_TI; RSR_S	JHI_Hv50k_2016_73712	2H	HORVU.MOREX.r2.2HG0088580	Q_73712_2H‐4	Phosphoinositide phospholipase C	0.000243
4	DW_FW_R_S; WCP_S; WCPTI	JHI_Hv50k_2016_121099	2H	HORVU.MOREX.r2.2HG0164040	Q_121099_2H‐5	RNA‐binding protein 39	0.000511
5	DW_FW_R_S; WCP_S	JHI_Hv50k_2016_164652	3H	HORVU.MOREX.r2.3HG0195930	Q_164652_3H‐4	Late embryogenesis abundant (LEA) hydroxyproline‐rich glycoprotein family	0.000770
6	RL_C; Ger_C	JHI_Hv50k_2016_277678	5H	HORVU.MOREX.r2.5HG0349750	Q_277678_5H‐10	Core‐2/I‐branching beta‐1,6‐N‐acetylglucosaminyltransferase family protein	0.002724
7	DW_C; FW_C	JHI_Hv50k_2016_253405	4H	HORVU.MOREX.r2.4HG0328180	Q_253405_4H‐3	Basic helix‐loop‐helix (bHLH) DNA‐binding superfamily protein	0.002865
8	DW_FW_R_C; WCP_C; RL_TI; FWTI	JHI_Hv50k_2016_177059	3H	HORVU.MOREX.r2.3HG0223920	Q_177059_3H‐6	Calmodulin, putative	0.002979
9	DW_FW_R_S; WCP_S	JHI_Hv50k_2016_28733	1H	HORVU.MOREX.r2.1HG0044990	Q_28733_1H‐3	Pentatricopeptide repeat (PPR) superfamily protein	0.00377
10	SL_TI; SL_S	JHI_Hv50k_2016_305720	5H	HORVU.MOREX.r2.5HG0395620	Q_305720_5H‐16	Protein FAR1‐RELATED SEQUENCE 5	0.01213

Abbreviations: Bon_p, Bonferroni threshold of significance at *p* ≤ 0.05 based on 32,995 SNPs; C, control; CL, coleoptile; DW, dry weight; DW/FW‐R, DW/FW ratio; FW, fresh weight; Ger%, germination percent; Hsp, Hordeum vulgare ssp. spontaneum (wild barley); RL, root length; RSR, root shoot ratio; S, salinity (seawater); SL: shoot length; TI: tolerance index; WCP, water content percent.

### 3.3 Candidate genes associated with seedling salinity tolerance in the HEB‐400 panel

A set of candidate genes associated with key physiological and morphological traits under both control and seawater stress conditions were identified and found to co‐localize with the detected QTLs. Among them, *HORVU.MOREX.r2.5HG0350240*, annotated as a peroxisome biogenesis protein 3‐2, was localized within Q_278421_5H‐1, a QTL reported for wide range of traits including DW to FW ratio (DW_FW_R_C and DW_FW_R_S), water content percent (WCP_C and WCP_S), fresh weight tolerance index (FW_TI), coleoptile length (CL_C and CL_S), shoot length (SL_C), root length (RL_C), and several TIs such as WCP_TI and RSR_C. This broad association suggests a potentially central regulatory role in multiple physiological processes under salinity stress (Table 2; Figure 5). Another key gene, *HORVU.MOREX.r2.2HG0178720*, annotated as cysteine proteinase inhibitor, was linked to Q_143769_2H‐3, a QTL related to salt TI, specifically WCP_TI and CL_TI, indicating its possible involvement in stress‐related growth modulation.

Similarly, *HORVU.MOREX.r2.2HG0088580*, annotated as phosphoinositide phospholipase C, was associated with root‐to‐shoot ratio TI and seawater stress (RSR_TI and RSR_S), pointing to its role in stress‐induced signaling pathways. The gene *HORVU.MOREX.r2.2HG0164040*, annotated as RNA‐binding protein 39, was linked to DW_FW_R_S, WCP_S, and WCP_TI, suggesting its contribution to posttranscriptional regulation under salt stress. In addition, *HORVU.MOREX.r2.3HG0195930*, a gene belonging to the late embryogenesis abundant hydroxyproline‐rich glycoprotein family, was also associated with DW_FW_R_S and WCP_S, supporting its well‐known function in cellular protection during dehydration and salt stress (Table 2; Table S5).

Further, *HORVU.MOREX.r2.5HG0349750*, annotated as a core‐2/I‐branching beta‐1,6‐N‐acetylglucosaminyltransferase family protein, was linked with RL_C and GER_C, suggesting a role in early developmental processes. The transcription factor *HORVU.MOREX.r2.4HG0328180*, a member of the basic helix‐loop‐helix (bHLH) DNA‐binding superfamily, was found to be associated with DW_C and FW_C, suggesting it implication in growth regulation under normal conditions. For their part, *HORVU.MOREX.r2.3HG0223920*, a putative calmodulin, showed associations with DW_FW_R_C, WCP_C, RL_TI, and FW_TI, highlighting its involvement in calcium‐mediated stress signaling. *HORVU.MOREX.r2.1HG0044990*, a pentatricopeptide repeat superfamily protein, was detected in association with DW_FW_R_S and WCP_S, reinforcing the significance of RNA processing proteins in stress responses. Lastly, *HORVU.MOREX.r2.5HG0395620*, annotated as protein FAR1‐RELATED SEQUENCE 5, was associated with SL under both TI and stress conditions (SL_TI and SL_S), indicating a possible role in light‐mediated growth regulation under saline environments.

These findings collectively underscore the multifaceted genetic basis of seawater tolerance in barley, highlighting genes involved in stress signaling, transcriptional and posttranscriptional regulation, growth, and developmental processes.

## 4 DISCUSSION

Our research focused on evaluating the seedling salinity tolerance of the HEB‐400 population. Unlike previous studies that simulated salinity stress using sodium chloride solutions (NaCl) alone (Munns & Tester, 2008; Roy et al., 2014), our study uses seawater to provide a more ecologically relevant and ionically complex representation of saline conditions. Seawater includes a mixture of Na^+^, Cl^−^, Mg^2^^+^, Ca^2^^+^, K^+^, and SO_4_^2^^−^ ions, better reflecting the challenges faced in natural saline soils and coastal agricultural environments (Plessis, 2023; De Souza et al., 2024). This approach enables us to investigate the effect of wild allele introgression on barley's performance under realistic salt stress (40% seawater), and to identify genetic factors contributing to improved seedling salt tolerance that may be overlooked in simplified NaCl‐based assay (Panta et al., 2014; Tester & Davenport, 2003).

Early seedling traits such as CL, RL, SL, FW, and DW play pivotal roles in determining barley's capacity to establish under salinity stress. Longer coleoptiles can enhance seedling emergence from deeper soil layers, which may be advantageous in saline soils where surface salt accumulation is common (Rebetzke et al., 2007). RL is particularly critical for salt tolerance, as longer and well‐branched roots can enhance access to less saline zones in the soil profile, support better water uptake, and facilitate spatial ion exclusion (Fan et al., 2015; Schneider et al., 2018; Shelden et al., 2016). Similarly, greater shoot elongation under salinity stress reflects the plant's ability to maintain cell expansion and turgor, which are linked to effective osmotic adjustment and ion compartmentalization (Munns & Tester, 2008; Shabala & Pottosin, 2014). Fresh and dry biomass accumulation are integrative indicators of overall vigor and stress resilience; genotypes with higher biomass under salinity often display improved photosynthetic performance and metabolic efficiency, despite ionic and osmotic constraints (James et al., 2008; Tavakkoli et al., 2012). The combined use of these morphological and physiological traits provides a reliable phenotypic framework for identifying salt‐tolerant genotypes via GWAS and supports their inclusion as selection criteria in pre‐breeding programs aimed at improving salinity tolerance (Ismail & Horie, 2017; Rajendran et al., 2009).

The HEB‐25 families displayed significant variation in their responses to seawater‐induced salinity stress at the seedling stage. Differences among families were evident across multiple growth parameters, including SL and RL, fresh and dry biomass, and RSRs (Figure 3; Tables S3 and S4). While some families (e.g., HEB10 and HEB24) showed less reduction in growth rates and biomass accumulation, others exhibited marked decreases under seawater treatment, highlighting varying levels of stress sensitivity (HEB25, HEB22, and HEB20). This differential response suggests that the introgression of wild alleles from *H. vulgare* ssp. *spontaneum* contributes to enhanced salinity tolerance in specific genetic backgrounds (Naz et al., 2014). This enhancement is due to the presence of stress‐adaptive alleles in the wild gene pool that were lost or underutilized during domestication, restoring or improving stress tolerance traits (Raza et al., 2025; Sayed et al., 2021). These findings underscore the potential of the HEB‐400 panel for identifying salt‐tolerant genotypes and dissecting the genetic basis of early‐stage stress adaptation.

Barley seeds exhibit a relatively high tolerance to environmental stresses, enabling them to maintain viability under adverse conditions. However, the germination and seedling emergence are among the most sensitive stages in the plant life cycle, particularly when exposed to abiotic stresses such as drought, salinity, and heat, which are becoming more frequent and severe (Baik & Ullrich, 2008; Nevo & Chen, 2010). Successful establishment during these stages is crucial for achieving a uniform crop stand and increase yield potential. Consequently, breeding programs aiming for climate resilience must focus on selecting genotypes that combine seed longevity with enhanced germination capacity and seedling vigor under abiotic stress conditions (Leroy et al., 2017; Mwando et al., 2020, 2021). Genetic variation in these traits through the exploration of wild alleles offers a valuable resource for developing barley cultivars that are better adapted to marginal environments and future climates scenarios (Varshney et al., 2017; Al‐Qadumii et al., 2025; Raza et al., 2025).

The outcomes of this study highlight the significant effect of seawater treatment on barley morphological traits, most notably increases in the DW to FW ratio (DW/FW‐R) under salt‐stress, suggesting that seawater‐treated plants lose water but retain a relatively higher proportion of dry matter (Munns & Tester, 2008; Munns & Gilliham, 2015). The higher median for DW/FW‐R under seawater treatment compared to control conditions suggests increased osmotic adaptation or water loss in response to salinity, consistent with previous studies linking this trait to plant adaptation under stress conditions. While traits such as SL, RL, and FW showed consistent reductions under seawater stress, other traits such as CL, RSR, and WCP evidenced less distinct changes. Reductions in seedling growth parameters under saline and osmotic stress have been previously reported in barley (Badr et al., 2025; Thabet et al., 2021). Such variation likely reflects stage‐ and trait‐specific responses to salinity stress, including seawater treatment, as has also been observed in other cereal crops (Ali et al., 2009; Roy et al., 2014; El Sabagh et al., 2021).

The absence of a significant difference in Ger% between control and saline stress suggests that seed germination in these genotypes is relatively unaffected. This resilience may be attributed to the protective role of the seed coat against salt intrusion, as well as inherent genetic tolerance mechanisms during early germination. Previous studies also reported no significant differences for these traits under saline and drought stress, where seed germination traits showed limited sensitivity across various barley genotypes (Ge et al., 2023; Pang et al., 2023; Ibrahim, 2016). Recently, Badr et al. (2025) analyzing a global barley panel reported similar %Ger levels under control versus osmotic stress but indicated an important reduction in the germination speed. Furthermore, the high heritability values for all traits indicate the probability and potential efficacy for selection in this panel (Saade et al., 2016; Pham et al., 2024).

In our study, several HEB‐25 families showed lower reductions in seedling growth traits under seawater stress conditions, indicating a high level of stress tolerance. Our results showed that, as a mean of all genotypes, certain HEB families harbor beneficial alleles from wild donors that enhance early‐stage stress tolerance beyond the elite genotype. Moreover, the dispersion in the data indicates extensive genotypic variation between genotypes from the same families (Figure 3). Some genotypes, for example, HEB_24_044, HEB_10_086, HEB_16_096, HEB_21_149, HEB_19_123, HEB_13_116, HEB_14_014, HEB_13_042, and HEB_24_066 demonstrated high tolerance under 40% seawater stress, maintaining stable Ger% and exhibiting the highest TIs for RL, SL, and/or DW compared to Barke (Tables S2 and S4).

Interestingly, the elite cultivar Barke consistently demonstrated intermediate to high performance under salinity stress, in agreement with previous findings under both field and controlled conditions (Saade et al., 2016; Wiegmann et al., 2019). This stable performance across diverse environments further supports its use as a reference genotype in genetic studies and breeding programs (Herzig et al., 2018). Notably, while this genotype outperformed an important proportion of the HEB lines, several introgression lines exhibited comparable or superior performance under salt stress (Table S3). This indicates that specific wild barley alleles can confer beneficial effects even when introgressed into an already high‐performing elite background (von Korff et al., 2005; Maurer et al., 2015).

The observed correlations between traits under control and seawater treatment conditions expose key relationships. For example, the highly positive correlations between RSR and WCP under both control and salinity conditions show that genotypes preserving a higher RSR also tolerate water content, a key factor for salinity tolerance (Thabet et al., 2021). For their part, the strong negative correlation between DW/FW ratio and WCP under seawater treatment highlights a trade‐off between biomass allocation and water retention in response to ionic and osmotic stress (Deinlein et al., 2014). This association could serve as a valuable indicator for identifying genetic traits linked to enhanced salt tolerance (Alsamadany et al., 2024).

In barley, GWAS has proven to be effective in identifying SNPs that are connected with salinity tolerance, helping researchers identify genes involved in key physiological processes such as ion homeostasis, osmotic regulation, and antioxidant defense (Fan et al., 2016; Alsamadany et al., 2024). GWAS enables the segmentation of genetic variation in barley populations, making it a valuable method for identifying natural variation in salinity tolerance (Sayed et al., 2022).

In our study, GWAS was conducted using a custom SAS‐based multi‐locus workflow incorporating subsampling, prediction error–based model selection, and cofactor correction to control for genetic background and reduce false positives. Owing to the NAM population design and the absence of strong population stratification, relatedness was modeled using an IBS matrix, prioritizing correction for genetic background effects (Vilhjálmsson & Nordborg, 2013). This approach has been previously validated and shown to yield results comparable to established methods such as FarmCPU (Liu et al., 2016., Dreissig et al., 2020; Schreiber et al., 2022). Through this analysis, we identified 60 QTLs across all seven chromosomes. Chromosome 5H showed the highest number (19), followed by 1H (15), 6H (eight), and 3H (seven). Chromosomes 2H, 4H, and 7H contained 6, 4, and 1 QTLs, respectively, highlighting genome‐wide variation for the studied traits (Figure 3). The higher number of QTLs detected in some traits under control conditions compared with seawater stress likely reflects that genetic variance for growth traits is more fully expressed in non‐stress conditions, whereas salinity imposes strong physiological constraints that reduce phenotypic variability and the detectability of allele effects. This phenomenon has been widely reported in cereals, where stresses such as salinity attenuate or mask QTL expression, leading to fewer significant associations under stress (Mickelbart et al., 2015; Morton et al., 2019). Furthermore, salt tolerance is typically governed by numerous small‐effect loci and strong genotype × environment interactions, which reduce GWAS power compared to control traits (Gupta & Huang, 2014; Cubillos et al., 2012). Similar reductions in QTL detection under stress have been observed in barley and wheat, supporting the idea that stress‐response pathways are more polygenic, environment‐dependent, and tightly regulated than those underlying plant growth in non‐stress conditions (Zhu et al., 2023; W. Wang et al., 2024). Notably, several markers associated with traits such as RL (RL_C, RL_TI) and shoot weight (WCP_S, WCP_C) were mapped to chromosomes 1H, 2H, and 5H, regions previously reported by Saade et al. (2016) and Maurer et al. (2015) as harboring key loci for abiotic stress tolerance. For instance, the marker JHI_Hv50k_2016_143769 on 2H, linked to WCP_TI, coincides with a QTL related to shoot biomass production under stress conditions, reinforcing the role of this genomic region in maintaining growth under salinity stress. For their part, markers JHI_Hv50k_2016_308562 (related to RSR_TI) and SCRI_RS_6793 (DW_FW_RTI) co‐localize with ion homeostasis genes reported by Saade et al. (2016). Specifically, JHI_Hv50k_2016_305720 (associated with SL_TI) overlaps with the shoot architecture QTLs identified by Maurer et al. (2015) and the candidate kinase gene *HORVU.MOREX.r2.5HG0395620*. This suggests a potential functional basis for the morphological adaptations observed. Novel markers, such as JHI_Hv50k_2016_465497, highlight unexplored genetic mechanisms for germination plasticity. On the other hand, our analysis revealed significant variation in the distribution of positive and negative effects of wild barley QTL alleles (wild alleles) on treatment outcomes across different genomic regions (Table S5).

The identification of candidate genes associated with physiological and morphological traits under control and seawater stress conditions provides valuable insights into the genetic basis of salt tolerance in this crop. Notably, *HORVU.MOREX.r2.5HG0350240* (encoding a peroxisome biogenesis protein 3‐2) exhibited broad involvement across a wide range of traits, suggesting its potential central role in regulating stress responses, possibly through reactive oxygen species (ROS) detoxification and peroxisomal signaling. Remarkably, this region overlaps with the salinity tolerance QTL cluster reported by Saade et al. (2016) who analyzed the salinity tolerance of the HEB‐25 panel under field conditions, suggesting its fundamental role in ionic homeostasis. Peroxisomes are dynamic organelles that play a critical role in abiotic stress responses by modulating ROS scavenging, lipid metabolism, and stress signal pathways (Sandalio et al., 2021; Su et al., 2019). The peroxisome biogenesis protein 3‐2 (PEX3‐2) is required for the proper formation and maintenance of peroxisomes. A promoter alternately positioned 315 bp upstream of the coding region possibly affects the binding of transcription factors, thereby altering the expression of PEX3‐2. This modulation in expression could influence peroxisome number or functionality, ultimately impacting processes such as fatty acid β‐oxidation and ROS sifting under salinity stress. The broad spectrum of trait associations identified in our analysis suggests that the observed cis‐regulatory modification may influence peroxisomal function, potentially playing a pivotal role in mediating plant adaptation to salinity stress (Ismail & Horie, 2017; Colebrook et al., 2014).

CL is a key trait influencing seedling emergence and establishment under saline conditions, as longer coleoptiles facilitate soil penetration and improve early vigor, particularly in stress‐affected soils (Rebetzke et al., 2007). The association of *HORVU.MOREX.r2.2HG0178720* (annotated as cysteine proteinase inhibitor) with CL_TI reported here suggests its role in growth modulation and protein turnover during salt stress adaptation. Other candidates genes such as *HORVU.MOREX.r2.2HG0088580* and *HORVU.MOREX.r2.3HG0223920* encoding a phosphoinositide phospholipase C and calmodulin, respectively, are known for their involvement in signal transduction, and their associations with TIs and root traits imply a potential contribution to root system architecture and functionality under stress (Li et al., 2017; Shen et al., 2020). Genes associated with LEA proteins (*HORVU.MOREX.r2.3HG0195930*) and RNA‐binding proteins (*HORVU.MOREX.r2.2HG0164040*) in traits such as water content and DW under salt conditions supports their roles in protecting against abiotic stress and posttranscriptional regulation (T. Wang et al., 2025). Moreover, candidates genes associated with bHLH transcription factors (*HORVU.MOREX.r2.4HG0328180*) and FAR1‐related proteins (*HORVU.MOREX.r2.5HG0395620*), both implicated in growth and light response, indicates potential cross‐talk between stress signaling and developmental pathways (Zhao et al., 2024; Dai et al., 2022). The diversity of gene functions and trait associations reported here underscores the complex and multigenic nature of salt tolerance, providing a foundation for functional validation and marker development in barley breeding programs.

The QTL Q_253405_4H‐3 is located 400 bp upstream of a gene that encodes a basic helix‐loop‐helix (bHLH) transcription factor. The bHLH proteins are well known for their role in controlling plant growth, development, and stress responses (Rajendran et al., 2009; Shabala & Pottosin, 2014). They bind to E‐box motifs in the promoters of target genes, thereby modulating gene expression networks that control processes such as cell elongation and hormonal signaling (Gao et al., 2024; Guo et al., 2015). The QTL associated with the bHLH gene, which is primarily linked to DW and FW traits, could influence the transcriptional regulation of this gene. This could adjust the expression levels of downstream targets involved in biomass accumulation and stress‐responsive pathways. Accordingly, this alternative could promote variations in plant growth under salinity stress by influencing both metabolic adjustments and cellular expansion.

To confirm the functional relevance of the QTLs and associated candidate genes identified in this study, further validation through independent studies is essential. Multi‐environment trials, including replicated greenhouse experiments and field trials on natural saline soils, will be critical to assess the stability and environmental responsiveness of QTL effects (Saade et al., 2016). Moreover, the development of near‐isogenic lines or heterozygous inbred families from HEB‐25 lines segregating at key QTL loci will allow precise phenotypic evaluation in a nearly uniform genetic background, facilitating the fine mapping of causal alleles (Maurer et al., 2015; Schmalenbach et al., 2009). Besides, functional characterization of candidate genes within QTL intervals may be pursued using targeted genome editing techniques such as CRISPR/Cas9, as demonstrated in barley for abiotic stress‐related traits (Kapusi et al., 2017). In parallel, gene expression profiling under salt stress conditions will help validate candidate genes based on their differential transcriptional regulation, particularly for transporters, signaling components, and transcription factors previously implicated in salt stress responses (Shavrukov, 2013). These integrative approaches will strengthen the causal links between marker loci and stress tolerance phenotypes.

The salt‐tolerant genotypes identified from the HEB‐400 panel, represent valuable pre‐breeding material for improving salinity tolerance in this crop. Interestingly, several genotypes that demonstrated superior tolerance at the seedling stage (e.g., HEB_15_113, HEB_19_115, HEB_15_051, and HEB_17_137) also exhibited higher grain yield and harvest index under saline field irrigation conditions compared to the elite cultivar Barke as reported by Saade et al. (2016). This overlap supports the idea that early‐stage salt tolerance may be predictive of improved performance at later developmental stages, reinforcing the potential of these genotypes for breeding barley adapted to saline environments. These lines can be incorporated into breeding programs through marker‐assisted backcrossing to introgress favorable alleles into regionally adapted elite cultivars while minimizing linkage drag (Saade et al., 2016; Schmalenbach et al., 2009; Mohamed et al., 2023). Moreover, the use of diagnostic markers linked to validated QTLs will expedite the pyramiding of multiple beneficial alleles for complex traits like salt tolerance. In addition, genomic selection models trained on phenotypic and genotypic data from the HEB‐25 panel may further enhance breeding efficiency by predicting salt tolerance potential in untested lines (Varshney et al., 2021). Importantly, several QTLs discovered in this study overlap with genomic regions previously associated with ion homeostasis and biomass maintenance under salinity stress (Fan et al., 2015), supporting their utility in breeding for enhanced performance under seawater intrusion and other saline environments. The integration of these wild‐derived alleles into pre‐breeding pipelines holds promise for improving resilience and yield stability in barley under increasingly challenging environmental conditions.

## 5 CONCLUSIONS

By leveraging genetic diversity within the NAM population HEB‐400, we identified tolerant germplasm and candidate genes that offer promising avenues for the development of stress‐resilient barley cultivars. These findings contribute to a deeper understanding of the genetic regulation of saline stress tolerance and hold practical relevance for breeding strategies to increase the challenges faced by climate change.

## AUTHOR CONTRIBUTIONS

**Matías Schierenbeck**: Data curation; formal analysis; investigation; methodology; software; validation; visualization; writing—original draft; writing—review and editing. **Radwa Y. Helmi**: Data curation; formal analysis; investigation; visualization; writing—review and editing. **Andreas Maurer**: Data curation; formal analysis; software; validation; visualization; writing—review and editing. **Rasha A. Tarawneh**: Data curation; investigation; writing—review and editing. **Doaa H. Ali**: Data curation; formal analysis; investigation; visualization; writing—review and editing. **Hannah M. Schneider**: Funding acquisition; supervision; writing—original draft; writing—review and editing. **Andreas Börner**: Conceptualization; funding acquisition; resources; supervision; writing—original draft. **Klaus Pillen**: Investigation; resources; supervision; writing—original draft. **Helmy M. Youssef**: Conceptualization; formal analysis; funding acquisition; investigation; methodology; project administration; supervision; writing—original draft; writing—review and editing.

## CONFLICT OF INTEREST STATEMENT

The authors declare no conflicts of interest.

## Supporting information




**Figure S1**. Principal Coordinate Analysis (PCoA) of the HEB‐400 population based on a genetic similarity matrix.
**Figure S2**. Linkage disequilibrium (LD) decay in the HEB‐400 population.
**Figure S3**. Pearson Correlation Coefficients of BLUEs values of the studied traits between (a) control, (b) sea‐water 40 and (c) tolerance index in 400 barley genotypes derived from the HEB‐25 population. The degree of significance for all correlations was P < 0.001. The color reflects the strength of the correlation. Black crosses indicate non‐significant correlations.
**Figure S4**. Manhattan plots showing the distribution of QTLs associated with, root/shoot length ratio (j‐l), seedling fresh weight (m) and germination percentage (n‐o) under control conditions, seawater conditions and tolerance index for the 400 barley HEB lines. QTLs are ordered based on Morex RefSeq2 (Monat et al., 2019). The red dashed line indicates the Bonferroni threshold of significance at 0.05 based on 32,995 SNPs (LOD ≥ 5.82).

**Table S1**. Summary statistics and ANOVA of seedling growth traits of HEB‐400 BC1S3 genotypes selected from the multi‐parental barley NAM population Halle Exotic Barley (HEB‐25) and evaluated under control and sea water stress conditions.
**Table S2**. Seedling growth parameters means for HEB‐400 genotypes and Barke under control and sea‐water stress conditions.
**Table S3**. Seedling growth parameters means for the 25 HEB families and Barke under control and sea‐water stress conditions.
**Table S4**. Pairwise multiple‐comparison analysis (LSD test) for key salinity‐responsive traits in the HEB‐400 population and the recurrent parent Barke. For each trait, the elite genotype Barke is highlighted in red. The ranking reflects the relative tolerance of genotypes for each trait.
**Table S5**. Significant markers and their associated candidate genes, LOD slcores, effect estimates and PVE values for seedling growth traits under seawater salinity stress in the HEB‐400 Panel. Multi‐trait QTLs are highlighted in green.

## Data Availability

Additional supporting information (phenotypic and GWAS results) can be found online in the Supporting Information section (Tables S1–S5). The genotype matrix is available at e!DAL (Maurer & Pillen, 2021).
