# Supplementary material for: Introgression of wild barley alleles improves seedlings salinity tolerance in the nested association mapping HEB‐400 population
Source: Plant Genome. 2026 Mar 15;19(1):e70217. doi: 10.1002/tpg2.70217 (PMC12989094; doi:10.1002/tpg2.70217)
Supplement: Supplementary file 1 — Figure S1. Principal Coordinate Analysis (PCoA) of the HEB‐400 population based on a genetic similarity matrix. Figure S2. Linkage disequilibrium (LD) decay in the HEB‐400 population. Figure S3. Pearson Correlation Coefficients of BLUEs values of the studied traits between (a) control, (b) sea‐water 40 and (c) tolerance index in 400 barley genotypes derived from the HEB‐25 population. The degree of significance for all correlations was P < 0.001. The color reflects the strength of the correlation. Black crosses indicate non‐significant correlations. Figure S4. Manhattan plots showing the distribution of QTLs associated with, root/shoot length ratio (j‐l), seedling fresh weight (m) and germination percentage (n‐o) under control conditions, seawater conditions and tolerance index for the 400 barley HEB lines. QTLs are ordered based on Morex RefSeq2 (Monat et al., 2019). The red dashed line indicates the Bonferroni threshold of significance at 0.05 based on 32,995 SNPs (LOD ≥ 5.82). [file TPG2-19-e70217-s001.docx]

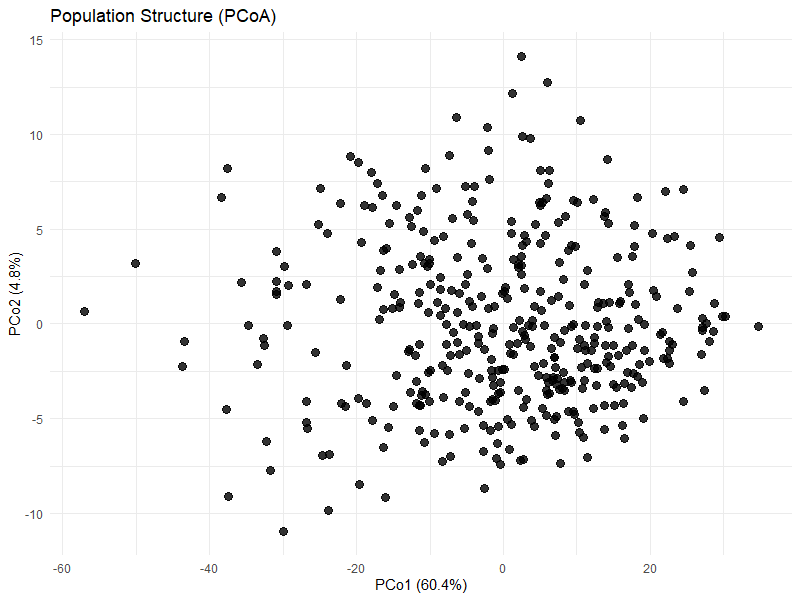


**Figure S1.** Principal Coordinate Analysis (PCoA) of the HEB-400 population based on a genetic similarity matrix.


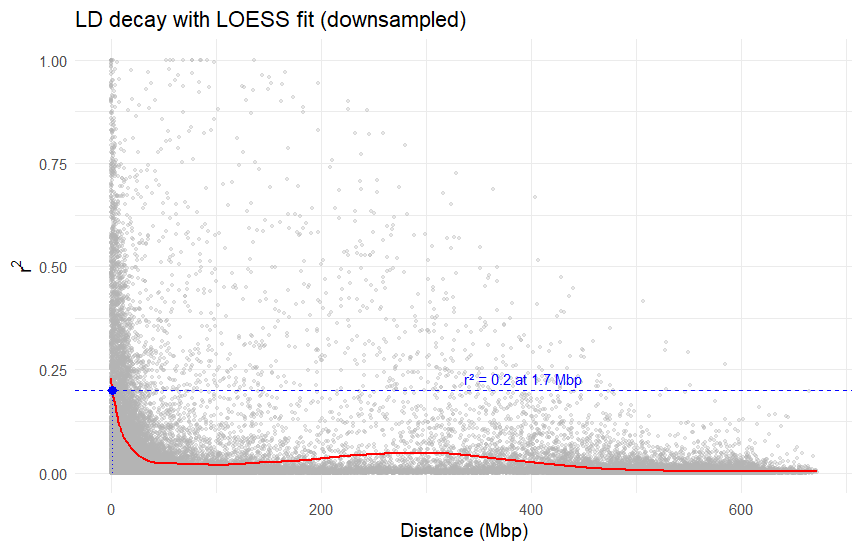


**Figure S2.** Linkage disequilibrium (LD) decay in the HEB-400 population


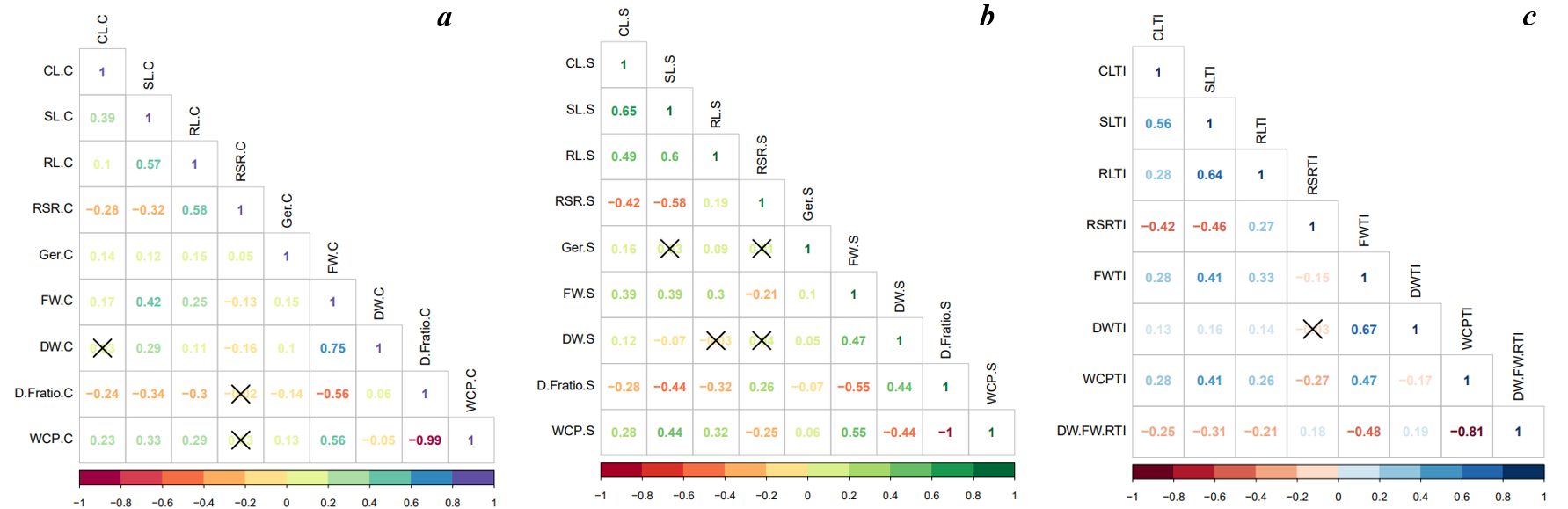


**Figure S3.** Pearson Correlation Coefficients of BLUEs values of the studied traits between (a) control, (b) sea-water 40 and (c) tolerance index in 400 barley genotypes derived from the HEB25 population. The degree of significance for all correlations was P<0.001. The color reflects the strength of the correlation. Black crosses indicate non-significant correlations.


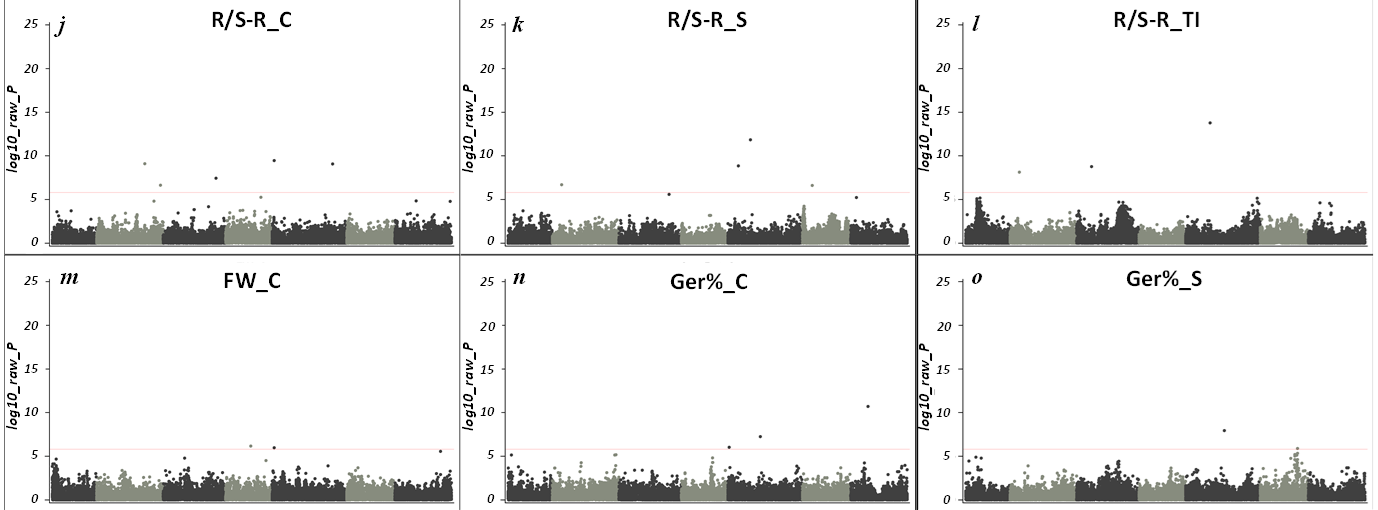


**Figure S4.** Manhattan plots showing the distribution of QTLs associated with, root/shoot length ratio (j-l), seedling fresh weight (m) and germination percentage (n-o) under control conditions, seawater conditions and tolerance index for the 400 barley HEB lines. QTLs are ordered based on Morex RefSeq2 (Monat et al. 2019). The red dashed line indicates the Bonferroni threshold of significance at 0.05 based on 32,995 SNPs (LOD≥ 5.82).
